# Leaf Organogenesis Improves Recovery of Solid Polyploid Shoots from Chimeric Southern Highbush Blueberry

**DOI:** 10.3390/biotech14020048

**Published:** 2025-06-12

**Authors:** Emily Walter, Akshaya Biswal, Peggy Ozias-Akins, Ye Chu

**Affiliations:** 1 Horticulture Department, The University of Georgia, Tifton Campus, 2356 Rainwater Road, Tifton, GA 31793, USA; emily.walter@uga.edu (E.W.); akbiswal@uga.edu (A.B.); pozias@uga.edu (P.O.-A.); 2 Institute of Plant Breeding, Genetics and Genomics, The Plant Center, The University of Georgia, Tifton Campus, 2356 Rainwater Road, Tifton, GA 31793, USA

**Keywords:** blueberry, mixoploid, chimera, adventitious shoot, axillary shoot, *Vaccinium*

## Abstract

Interspecific and intersectional crosses have introduced valuable genetic traits for blueberry (*Vaccinium* sect. *Cyanococcus*) cultivar improvement. Introgression from *Vaccinium* species at the diploid, tetraploid, and hexaploid levels has been found in cultivated blueberries. Continued efforts to integrate wild blueberry genetic resources into blueberry breeding are essential to broaden the genetic diversity of cultivated blueberries. However, performing heteroploid crosses among *Vaccinium* species is challenging. Polyploid induction through tissue culture has been useful in bridging ploidy barriers. Mixoploid or chimeric shoots often are produced, along with solid polyploid mutants. These chimeras are mostly discarded because of their genome instability and the difficulty in identifying periclinal mutants carrying germline mutations. Since induced polyploidy in blueberries often results in a low frequency of solid mutant lines, it is important to recover solid polyploids through chimera dissociation. In this study, two vegetative propagation methods, i.e., axillary and adventitious shoot induction, were evaluated for their efficiency in chimera dissociation. Significantly higher rates of chimera dissociation were found in adventitious shoot induction compared to axillary shoot induction. Approximately 89% and 82% of the adventitious shoots induced from mixoploid lines 145.11 and 169.40 were solid polyploids, respectively, whereas only 25% and 53% of solid polyploids were recovered through axillary shoot induction in these lines. Effective chimera dissociation provides useful and stable genetic materials to enhance blueberry breeding.

## 1. Introduction

True blueberries (*Vaccinium* sect. *Cyanococcus*) are native to North America and consist of 9 to 24 species across the diploid, tetraploid, and hexaploid levels, depending on the system of species classification [1]. These *Vaccinium* species are found in highly diverse environments across northeastern United States, resulting in a broad range of genetic diversity [2] (Vander Kloet 1988). This rich genetic resource has been utilized for blueberry domestication and breeding through intraspecific, interspecific, and intersectional crosses since the late 1800s [3,4,5]. Lowbush (2*n* = 4*x* = 48; *V. augustifolium*), northern highbush (2*n* = 4*x* = 48; *V. corymbosum),* southern highbush (2*n* = 4*x* = 48; *V. interspecific*), rabbiteye (2*n* = 6*x* = 72; *V. virgatum*), and half-high bush (2*n* = 4*x* = 48) (produced from interspecific crosses between lowbush and highbush blueberries) are grown in the US for commercial production [6]. Tetraploid species—*V. corymbosum* and *V. augustifolium*—and hexaploid *V. virgatum* are the primary genetic bases for these cultivated blueberries. Simple sequence repeat (SSR) markers and the pedigree information of blueberry cultivars are typically used to determine the contribution of wild blueberries to the genetic diversity in cultivated blueberries [7,8,9]. Phylogenetic analyses revealed that southern highbush blueberries form a distinct group from northern highbush blueberries [7]. Introgression from seven *Vaccinium* species contributed to the genetic makeup of SHB blueberries. Gene flow among diploid and tetraploid *Vaccinium* species was identified through genotyping by sequencing analysis (GBS), further confirming the role of interspecific and intersectional introgression in blueberry cultivar development [10].

Although introgression from wild blueberries plays an important role in blueberry breeding, crosses among *Vaccinium* species at multiple ploidy levels have differing levels of success [11]. Homoploid crosses are prolific and have produced vigorous progenies in intraspecific crosses [12,13]. Likewise, homoploid interspecific and intersectional crosses among *Vaccinium* species have also produced viable hybrids, although the number of progenies was reduced [14,15,16,17,18,19]. On the contrary, heteroploid crosses are challenging. Strong triploid blocks from crosses between diploid and tetraploid species have resulted in very low numbers of hybrid progenies [20,21], and these progenies could not be used for any backcrosses due to pollen sterility [22,23]. However, diploid species that can produce 2*n* gametes—such as Florida 4B, a diploid *V. darrowii* clone—were successfully crossed with tetraploid highbush blueberries and contributed to the adaptation of SHB to the low-chilling growing environment [24,25,26]. Although heteroploid crosses between tetraploid highbush and hexaploid rabbiteye produced partially fertile pentaploid hybrids, crossing success rate and progeny fertility were highly dependent on the parental genotype combinations [27].

To overcome the ploidy barrier, artificial polyploid induction has been performed to double the ploidy levels of diploid species and triploid hybrids [28]. For instance, *V. arboreum* (2*n* = 2*x* = 24, *V.* sect. *Batodendron*) and *V. stamineum* (2*n* = 2*x* = 24, *V.* sect. *Polycodium*) were not cross compatible with cultivated blueberries [29,30], whereas colchicine-induced tetraploid *V. arboreum* and *V. stamineum* could produce viable and fertile intersectional hybrids when crossed with highbush blueberries [31,32]. Recovery rates of solid polyploids from polyploid induction ranged from 1 to 10% [16,30,33,34]. Additionally, some of the chromosomally doubled new genetic materials were less vigorous than the wild types and were lost during vegetative propagation [32]. Besides the desired solid polyploids, polyploid induction often produces mixoploid shoots, i.e., chimeras. Chimeras are tissues or shoots containing cells with different genotypes that frequently result from mutation [35,36]. The apical meristem of a plant shoot is organized into three histogenic layers [37]. Layer I develops into epidermal tissue; layer II consists of subepidermal tissue such as leaf, anthers, and ovules; and layer III develops into vascular tissues such as the xylem and phloem. Depending on the location and ratio of the mutant and normal cells in the apical meristem, there are three types of chimeric plants, i.e., periclinal, mericlinal, and sectorial chimeras. Periclinal chimeras are derived from a mutant cell whose further division covers an entire layer of the apical dome and produces a whole layer of mutant tissue. Periclinal chimeras can be maintained through vegetative propagation [38]. Mericlinal chimeras only have a part of one meristem layer developed from a mutant cell; the remaining portion of the cell layer remains wild type. Sectorial chimeras have more than one cell layer comprising mutant cells. Neither mericlinal nor sectorial chimeras are stable, and hence they lose the mutation through vegetative propagation. Previously, periclinally chimeric *V. darrowii* (2*n* = 2*x* = 24) plants—with chromosomal doubling occurring at the L2 histogenic layer—were identified by their enlarged pollen grains [18]. Although these periclinal chimeras are useful for breeding, it takes at least two years before their pollen grains become available for chimeric screening due to the long juvenile stage of blueberry bushes. In addition, screening for stable periclinal chimeras by morphological traits is tedious. Therefore, chimeras are mostly discarded because of the instability of their polyploidy levels and the difficulty in screening [39,40].

The recovery of solid polyploids by dissociating chimeric cells in mixoploid plants is highly desirable, as the recovery rate of solid polyploids is typically low and valuable new polyploids can be lost during subsequent propagation. In vitro techniques have been used in other species to recover solid polyploids. Two types of shoot induction are common in plant tissue culture, i.e., axillary and adventitious shoot induction. Axillary shoots are induced from existing axillary buds, whereas adventitious shoots are developed from non-meristematic tissue such as leaves. Multiple subcultures from mixoploid nodal segments of cassava (*Manihot esculenta*) increased the recovery of solid tetraploid [41]. Solid polyploid shoots were recovered from mixoploid leaf organogenesis in hops (*Humulus lupulus*) [42] and *Astragalus membranaceus* [43]. Bananas (*Musa* spp.) are generally propagated through shoot tip culture, multi-shoot tip culture, and corm slice culture. Among these three propagation systems, corm slice culture was found to be the most efficient in chimera dissociation [44]. For blueberries, no efforts to recover solid polyploids from chimeras have been reported; however, axillary shoot production from existing vegetative meristem using single-node segments is well established [45,46,47,48,49,50,51,52,53,54,55,56,57,58,59,60,61]. Adventitious shoot regeneration from leaf explants without pre-existing meristems has also been reported [62,63,64,65]. Evaluating these tissue culture systems for the recovery of solid polyploids from mixoploid shoots will inform their effectiveness in chimera dissociation in blueberries. Therefore, the objective of this study is to determine the efficiency of solid polyploid recovery from chimeras using axillary and adventitious shoot propagation systems. Effective chimera dissociation will increase the production of stable polyploids amenable for clonal propagation and breeding.

## 2. Materials and Methods

### 2.1. Plant Materials

Mixoploid mutants from southern highbush blueberry cultivars ‘Rebel’ [66] and ‘Emerald’ [67] were recovered from colchicine-treated plantlets through tissue culture. The medium and growth regulators were selected based on previous studies on tissue culture of *Vaccinium* species [53,68,69,70,71]. Briefly, axillary shoots were micropropagated from single-node stem segments in Woody Plant Medium 1 (WPM1) (Table 1). Elongated shoots of at least 2 cm in length were harvested and defoliated under aseptic conditions. Defoliated shoot stem explants were immersed in 0.02% or 0.2% colchicine solution and rocked at 30 rpm on an UltraRocker rocking platform (Bio-Rad Laboratories, Hercules, CA, USA) for 48 h in the dark at room temperature. These colchicine concentrations were previously reported to induce polyploidy in *Vaccinium* species [16,31,72]. The effectiveness of these colchicine concentrations is detailed in a separate study (manuscript in preparation). The explants were washed three times with sterilized deionized water, segmented into single-node segments, and then cultured on WPM1 medium until ploidy analysis. Mixoploid mutants were recruited for this study based on the flowcytometry analysis described below.

### 2.2. Flowcytometry Analysis

Two to three leaves were dissected from each regenerated plantlet and pooled for ploidy level determination. The protocol from the CyStain^®^ PI Absolute P staining kit (Sysmex Partec GmbH, Görlitz, Germany) was modified to accommodate 5–10 mg of the tissue harvested from the shoot explants. Briefly, equal amounts of leaf tissue from the target plantlet and a diploid wild blueberry *V. fuscatum* (2*n* = 2*x* = 24) plant were chopped together—using a double-edged razor blade—in 60 µL of extraction buffer for 30 to 60 s in a Petri dish chilled on ice. Next, 500 µL of working staining solution—consisting of 0.5 µL staining buffer, 3 µL propidium iodide (PI) dye, 1.5 µL of RNase, and 495 µL of water—was prepared using the reagents provided in the kit. The working solution was then added to the nuclei extract and incubated for 60 s. The PI-labeled nuclei solution was filtered through a 30 µm CellTrics filter (Sysmex Partec GmbH) and incubated on ice for 10 min. The samples were then run through the Attune NxT Acoustic Focusing Cytometer (ThermoFisher, Scientific, Walthman, MA, USA). The nuclei signal from *V. fuscatum* served as the internal control to derive the ploidy level of the target tissue. A minimum nuclei count of 2000 and an X-mean confidence interval less than 5% were included in ploidy determination using the following formula:

$$Target\;polyploid\;level=\frac{X\_mean\;of\;target}{X\_mean\;of\;V.\;fuscatum\;}*2$$

It should be noted that the X-mean value is the mean propidium iodide (PI) fluorescence signal emitted by the PI-labeled nuclei. This value indicates the fluorescence intensity of the target nuclei.

**Figure 1:**
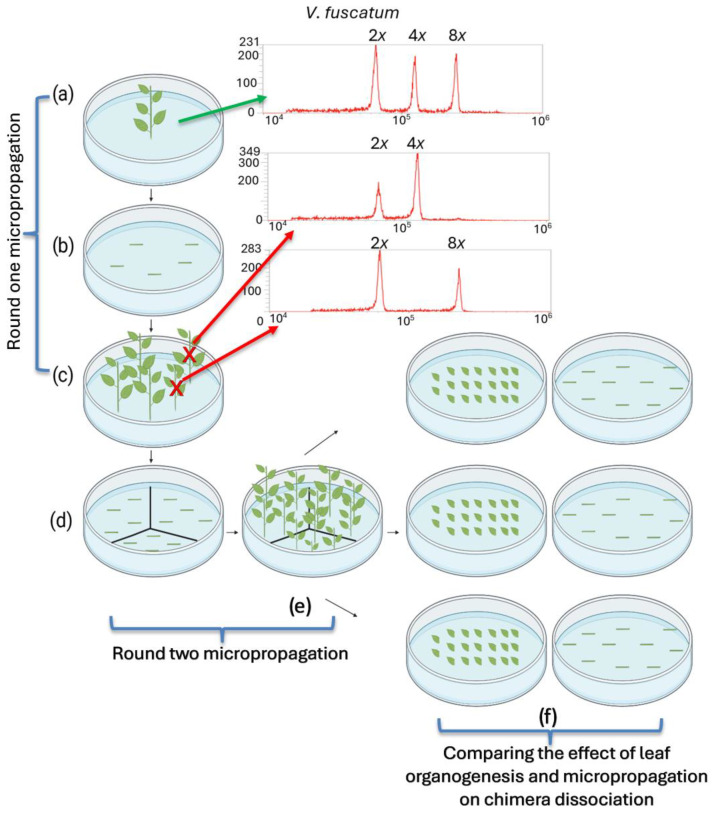
Diagram of a mixoploid mutant line propagated for testing the effects of leaf organogenesis and axillary shoot micropropagation on solid polyploid recovery. (**a**) A mixoploid mutant plantlet recovered from colchicine treatment was used for this study. The green arrow points to the flow cytometry plot showing three distinct peaks detected from the PI-labeled mixture of nuclei extracted from this plantlet and the *V. fuscatum* control. The left peak is the 2C nuclei signal from the diploid *V. fuscatum* control; the middle and right peaks are the tetraploid and octoploid 2C signals from the mutant line, indicating its mixoploid level. (**b**) Single-node stem segments from the mixoploid plantlet were cultured to generate more axillary shoots through micropropagation. (**c**) The ploidy levels of the propagated stems were confirmed through flow cytometry analysis. If the new shoot was a tetraploid or an octoploid, as indicated by the red arrows, it was excluded from further culture. (**a**–**c**) depict the first round of micropropagation of the mutant line. (**d**) The confirmed mixoploid plantlets were micropropagated for a second round to obtain enough explant materials, as illustrated in (**e**,**f**). Leaves and single-node stems were dissected to evaluate the effect of regeneration methods on chimera dissociation. The leaf tissue formed 20 leaves per Petri dish for adventitious shoot regeneration through leaf organogenesis. The corresponding 10 stems per Petri dish were cultured for axillary shoot induction through micropropagation. The figure was created with BioRender (https://biorender.com, accessed on 12 March 2025).

Mixoploid Explant Tissue Culture:

Mixoploid mutant lines from ‘Rebel’ and ‘Emerald’, recovered from in vitro colchicine treatment, were micropropagated from single-node segments for the first round to generate sufficient materials for experimentation (Figure 1a–c). Each new shoot was treated as an independent regeneration unit for polyploidy determination. The ploidy level of the explants from the first round of micropropagation were tested by flow cytometry analysis. Shoots at either the tetraploid or octoploid level were excluded from further study (Figure 1c). The second round of micropropagation was performed for the selected mutant lines to increase the number of explants (Figure 1d,e). Cultures of single-node segments from the mixoploid plantlets were initiated on WPM2 (10 stem segments/plate) and then sub-cultured into WPM3 after three weeks (Table 1). To compare the efficiency of chimera dissociation, adventitious shoots were induced from leaf tissues of mixoploids. Briefly, twenty leaves (without petioles) were placed on abaxial side down in WPM4 media in a Petri dish (Figure 1f) and were sub-cultured into WPM5 media after three weeks (Table 1). Each Petri dish was considered as a biological replication, and three biological replications were included for each chimeric mutant line. Afterwards, the explants from both leaf and stem nodes were maintained in WPM1, which contained only 0.5 mg/L zeatin. Tissue culture plantlets were grown in the I-66LLVL growth chamber (Percival Scientific, Perry, IA, USA) set at 26 °C, with a 16/8 h light/dark cycle and photon flux density of 40–50 µmol/m^2^/s.

### 2.3. Explant Measurements

Images of the regenerated polyploid explants were collected using a digital microscope (Moysuwe, https://www.amazon.com/MOYSUWE-Microscope-Magnifier-Soldering-Compatible/dp/B0CB2J33SB?ref_=ast_sto_dp, accessed on 29 May 2025) and exported to APS Assess 2.0 (The American Phytopathological Society, St. Paul, MN, USA) for stem width measurement. A line 10 mm in length was drawn on the background of each image to serve as a scale for the physical size of each shoot.

### 2.4. Rooting of Plantlets

Plantlets 2 to 3 cm in length were harvested from tissue culture and planted in a 72-cell propagation tray (https://www.bootstrapfarmer.com) filled with a water-saturated pine bark medium. To encourage root formation, the propagation tray was covered with a clear cover that was clamped with clips to maintain the high humidity. The propagation tray was kept in the greenhouse with a temperature ranging from 18 to 28 °C. The cover of the tray was removed after root formation. The plantlets were watered and fertilized as needed with a slow-release 10-10-10 fertilizer (Scotts Miracle-Gro Company, Marysville, OH, USA).

### 2.5. Statistical Analysis

Student’s *t*-test was performed to determine if there was a statistically significant difference between the two shoot culture conditions in terms of the rate of shoot regeneration and chimera dissociation. Normality tests were performed using the Shapiro–Wilk test, and the datasets were found to be normally distributed. Shoot width among explants of three ploidy levels was analyzed using analysis of variance (ANOVA) and Tukey’s honestly significant difference (HSD) test, performed with the SAS 9.4 software (SAS Institute Inc., Cary, NC, USA). Post hoc analysis was performed when the genotype effect was found to be significant based on the ANOVA. Statistically significant differences were considered at *p* < 0.05.

## 3. Results and Discussion

Thirteen mixoploid mutant lines—four recovered from ‘Emerald’ and nine from ‘Rebel’—were identified during the initial screening of the axillary shoots propagated from colchicine-treated stem tissue by flow cytometry analysis (Table 2). These lines were micropropagated through single-node stem culture for the first round (Figure 1a–c) and yielded 66 explants, with 2 to 18 explants per mutant line. Flow cytometry screening of these explants indicated that 35 explants remained mixoploid; 2 explants were octoploid; and 29 were tetraploid. The average mixoploid retention rate was 63% and solid polyploid recovery rate was 37%. Interestingly, in all nine mutant lines that produced solid polyploids, only one mutant line, 143.13 from ‘Rebel’, produced axillary shoots with all three ploidy types (octoploid, tetraploid, and mixoploid). The rest produced only one type of solid polyploid, i.e., either octoploid or tetraploid, not both.

**Figure 2:**
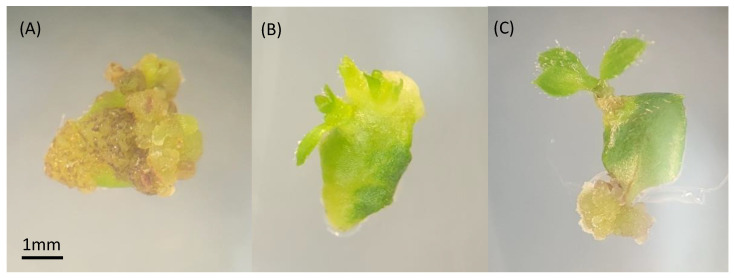
Leaf tissue with callus formation (**A**), emerging shoots (**B**), and elongated shoot (**C**) from organogenesis 48 DAT.

**Figure 3:**
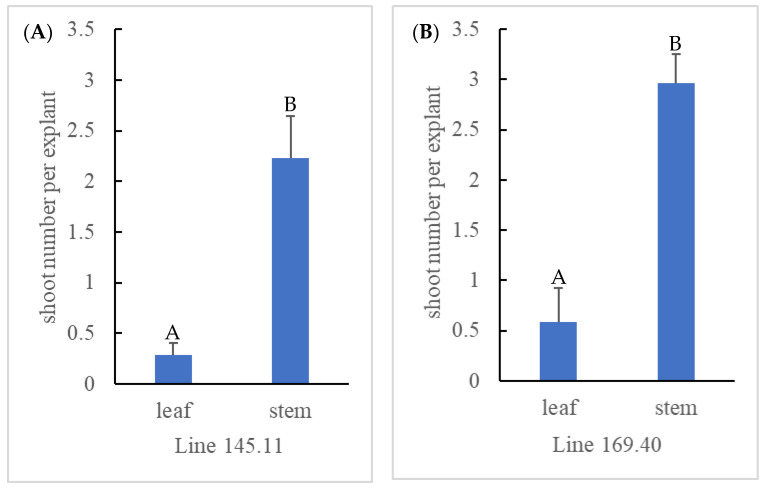
Comparison of number of shoots regenerated per explant from mixoploid mutant lines 145.11 (**A**) and 169.40 (**B**) through leaf organogenesis and single-node stem cultures. Statistically significant differences at *p* < 0.05 between the two tissue culture methods are denoted with different letters on top of bar graph.

To compare the efficiency of solid polyploid recovery between axillary and adventitious shoot induction, mixoploid mutant lines 145.11 and 169.40 from ‘Emerald’ and 175.35 and 175.40 from ‘Rebel’ were micropropagated for a second round (Figure 1d,e) to establish three plates each for adventitious (n = 20 per plate) and axillary (n = 10 per plate) shoot induction. None of the ‘Rebel’ mutant lines produced any adventitious shoots, probably due to inappropriate medium formulation, suboptimal growth hormone concentrations, or the light and temperature conditions of the tissue culture chamber. No further data were collected from these two lines. On the other hand, the two ‘Emerald’ mutant lines were responsive to both shoot induction protocols. In the process of adventitious shoot induction through leaf organogenesis, various morphological responses were observed among the mutant lines (Figure 2). Leaf tissue with emerging shoots (Figure 2B) and elongated adventitious shoots (Figure 2C) subsequently produced shoot explants for flow cytometry analysis. Over half the leaf tissue formed callus and failed to produce any adventitious shoots (Figure 2A). Zeatin levels in the medium were reduced to encourage shoot formation rather than callus formation. An average of 0.28 and 0.58 adventitious shoots per leaf explant were produced from leaf organogenesis for lines 145.11 and 169.40, respectively. In the meantime, axillary shoot induction from single-node stem explants produced an average of 2.23 and 2.96 shoots per stem from lines 145.11 and l69.40, respectively. The difference between the two shoot induction methods for both mutant lines was statistically significant (*p* < 0.05) (Figure 3). Supplementing zeatin between 0.5 and 2 mg/L to the culture medium was shown to be effective in promoting shoot production from single-node segments in highbush blueberries [52,53]. In our experiment, media supplemented with 0.5 to 3 mg/L zeatin were effective in shoot induction from single-node segments. For leaf organogenesis, 3 mg/L zeatin plus 0.5 mg/L IBA was found to be effective in shoot regeneration for highbush and rabbiteye blueberries [73]. Media supplemented with 0.2 to 1 mg/L thidiazuron (TDZ) and 0.2 to 0.5 mg/L 1-naphthaleneacetic acid (NAA) reportedly induce adventitious shoots from highbush blueberries [69]. We evaluated both zeatin/IBA and TDZ/NAA leaf organogenesis media for adventitious shoot production with wildtype ‘Emerald’ in a preliminary study. The medium supplemented with 1 mg/L TDZ and 0.5 mg/L NAA failed to produce shoots, whereas the medium with 3 mg/L zeatin and 0.5 mg/IBA produced shoots. This is aligned with the previous findings that the effectiveness of shoot induction is genotype dependent [68]. Several factors such as genotype of the explant, combination of the culture medium and growth regulators, tissue culture growing conditions, and the developmental stages of the explant have been suggested to affect the outcome of leaf organogenesis [68]. Therefore, the mixoploid leaf tissues were cultured on the latter medium for shoot induction. Although the mixoploid ‘Emerald’ lines contained mutant cells at the octoploid level, both the leaf and stem tissues from the mutant lines were responsive to shoot induction, similar to the wildtype ‘Emerald.’

**Figure 4:**
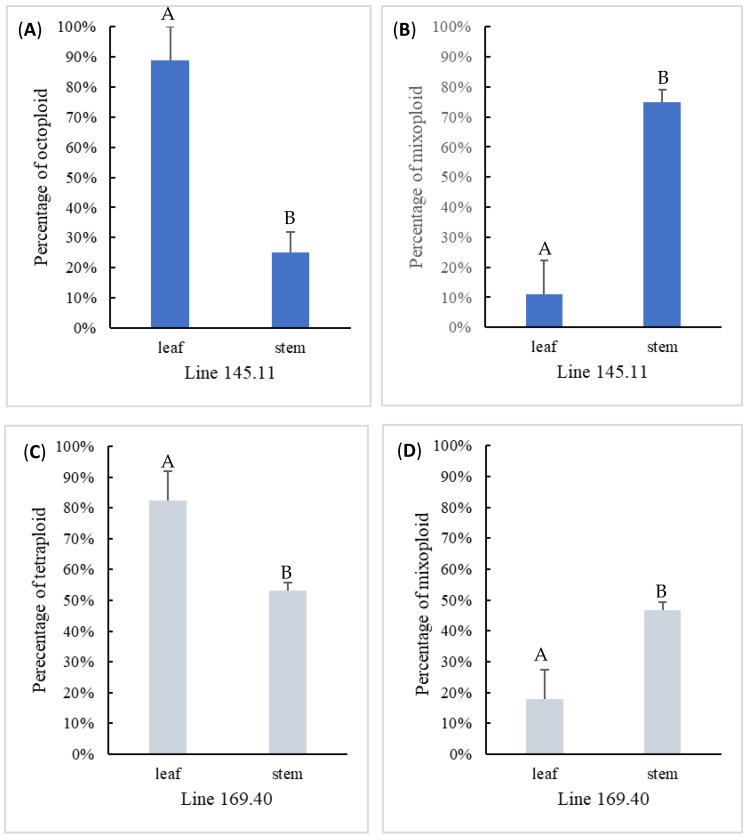
Comparison of two tissue culture methods in generating solid and mixoploid shoots for mixoploid mutant lines 145.11 (**A**,**B**) and 169.40 (**C**,**D**). Statistically significant differences at *p* < 0.05 between the two tissue culture methods are denoted with different letters on top of bar graph.

Two ploidy levels were detected among the regenerated shoot explants from both mixoploid mutant lines regardless of the method of shoot regeneration. Mutant line 145.11 produced octoploids and mixoploids, whereas line 169.40 produced tetraploids and mixoploids (Figure 4). The distribution of polyploid shoots among the two mutant lines was consistent with the screening results during the first round of propagation (Table 2). The percentage of solid polyploid (i.e., octoploid and tetraploid) adventitious shoots induced from leaf organogenesis was 89% and 82% for mutant lines 145.11 and 169.40, respectively, which was signficantly higher than those from the axillary shoots induced by micropropagation (25% and 53%, respectively). Since both lines only produced shoots of one type of solid polyploid or mixoploid, the mixoploid retention rate was significantly lower in adventitous explants than in axillary explants.

**Figure 5:**
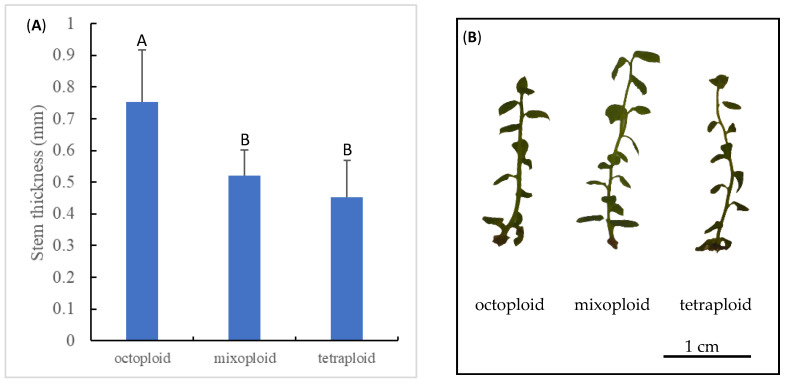
Stem thickness of regenerated octoploid, mixoploid, and tetraploid shoots (**A**) and representative images of shoots at the three ploidy levels (**B**). Statistically significant differences at *p* < 0.05 are denoted with different letters on top of bar graph.

Polyploidy is known to result in the enlargement of plant organs, otherwise known as the ‘giga’ effect [74]. To confirm chromosomal doubling in the octoploid explants, the stem thickness of the explants was measured. Consistent with the previous reports [72,75], stems from the colchicine-induced octoploid ‘Emerald’ shoots were significantly thicker than stems from the mixoploid and tetraploid ‘Emerald’ shoots (Figure 5).

Tissue culture allows for the generation of plants from a small number of cells; therefore, it is possible to separate mutants from wild-type cell types in a chimera and establish solid polyploid plants. Since axillary shoot induction produces new shoots from the existing axillary shoot meristem, the cells are already predestined to develop into new shoots. Therefore, axillary shoot formation through micropropagation tends to maintain the cell layer arrangement of the original meristem and has a high chance of retaining the chimeric status of periclinal chimeras [76]. On the other hand, in the process of adventitous shoot induction, non-meristemic cells are redirected to become meristemic, triggered by the high cytokinin (zeatin) and low auxin (IBA) concentrations in the culture medium. During leaf organogenesis, leaf cells in the mixoploids are redirected for meristemic shoot formation. Therefore, in theory, adventitious shoots can arise from any histogenic layer [36], offering a high likelihood of chimera dissociation and the recovery of either solid tetraploids (as line 169.40) or solid octoploids from the mixoploid plants (as line 154.11). Previously, adventitious shoot regeneration was reported to be efficient in dissociating tobacco chimeras [77]. Our results support these findings, as adventitious shoot regeneration resulted in a significantly higher rate of solid polyploid recovery than axillary shoot induction. However, the two chimeric mutants from ‘Rebel’ failed to produce any adventitious shoots, which may stem from the limited regeneration competency of mixoploid ‘Rebel’ mutant lines in the leaf organogenesis medium. Responsiveness to shoot induction medium is known to be genotype dependent in blueberries [68]. Optimizing the cytokinin source and levels for adventitious shoot production in ‘Rebel’ chimeras may provide the opportunity to assess the efficacy of chimera dissociation in these mutant lines. Five plantlets, each at the tetraploid and octoploid levels from both lines, were rooted in the propagation tray after forty days. All the plantlets successfully rooted, consistent with previous findings that ex vitro rooting of tissue culture-derived shoots is highly successful in blueberries [70,78].

## 4. Conclusions

Blueberries are an outcrossing species with inbreeding depression [24]. Broadening the genetic base through interspecific and intersectional hybridization is imperative to maintain the fitness and resilience of cultivated blueberries. However, these efforts often encounter ploidy barriers, resulting in failed crosses or infertile progenies. Polyploidy induction is an important tool for bridging the ploidy barrier in blueberry breeding. In the process of polyploidy induction, chimeric mutant lines are mostly discarded due to their genome instability. In this study, we endeavored to recover solid polyploids through chimera dissociation. Chimeric southern highbush blueberry explants from colchicine-treated ‘Emerald’ were cultured on leaf organogenesis and single-node micropropagation media. Significantly higher rates of chimera dissociation were found when the mutant lines were cultured within the leaf organogenesis medium. Recovery of solid polyploids from colchicine-induced polyploidization provides valuable stable genetic material for blueberry breeding.

## Figures and Tables

**Table 1:** Media Preparation *.

Media Name	Composition
Woody Plant Medium 1 (WPM1)	WPM (1x)**,** 3% Sucrose (*w*/*v*), 0.8% Agar and 0.5 mg/L zeatin, pH 5.2
WPM2	WPM (1x)**,** 3% Sucrose (*w*/*v*), 0.8% Agar, 3 mg/L zeatin and 0.5 mg/L IBA (Indole-3-butyric acid), pH 5.2
WPM3	WPM (1x)**,** 3% Sucrose (*w*/*v*), 0.8% Agar and 1 mg/L zeatin, pH 5.2
WPM4	WPM (1x)**,** 3% Sucrose (*w*/*v*), 0.8% Agar, 2 mg/L zeatin and 0.5 mg/L IBA, pH 5.2
WPM5	WPM (1x)**,** 3% Sucrose (*w*/*v*), 0.8% Agar, 1 mg/L zeatin and 0.5 mg/L IBA, pH 5.2

* The pH of all media was adjusted to 5.2 by 1N NaOH. All media were sterilized by autoclaving at 121 °C for 20 min. WPM (Phytotech, Lenexa, KS, USA), Sucrose (Aldon, Avon, NY, USA), Agar (Sigma Aldrich, St. Louis, MO, USA), zeatin and IBA (Phytotech).

**Table 2:** Ploidy levels of axillary shoots regenerated from first round of micropropagation (Figure 1a–c).

Genotype	Mutant Lines	Screened Explant	Octoploid	Tetraploid	Mixoploid	% Solid Polyploid	% Mixoploid
Emerald	145.11	4	1	0	3	25%	75%
Emerald	169.40	5	0	1	4	20%	80%
Emerald	173.68	3	0	2	1	67%	33%
Emerald	187.91	4	0	3	1	75%	25%
Rebel	143.11	3	0	2	1	67%	33%
Rebel	143.13	18	1	14	3	83%	17%
Rebel	143.15	10	0	3	7	30%	70%
Rebel	175.35	2	0	0	2	0%	100%
Rebel	175.40	2	0	0	2	0%	100%
Rebel	178.57	5	0	0	5	0%	100%
Rebel	179.68	3	0	0	3	0%	100%
Rebel	188.95	4	0	3	1	75%	25%
Rebel	194.156	3	0	1	2	33%	67%
	total	66	2	29	35		
	average	5.1	0.2	2.2	2.7	37%	63%

## Data Availability

The original contributions presented in this study are included in the article. Further inquiries can be directed to the corresponding author.

## References

[1] Fritsch P.W., Crowl A.A., Ashrafi H., Manos P.S. (2024). Systematics and evolution of *Vaccinium* sect. Cyanococcus (Ericaceae): Progress and prospects. Rhodora.

[2] Vander Kloet S.P. (1988). The Genus Vaccinium in North America.

[3] Coville F.V. (1910). Experiments in Blueberry Culture.

[4] Longley A. (1927). Chromosomes in *Vaccinium*. Science.

[5] Coville F.V. (1927). Blueberry chromosomes. Science.

[6] Hancock J.F. (2008). Temperate Fruit Crop Breeding: Germplasm to Genomics.

[7] Brevis P.A., Bassil N.V., Ballington J.R., Hancock J.F. (2008). Impact of wide hybridization on highbush blueberry breeding. J. Am. Soc. Hort. Sci..

[8] Boches P., Bassil N.V., Rowland L. (2006). Genetic diversity in the highbush blueberry evaluated with microsatellite markers. J. Am. Soc. Hort. Sci..

[9] Bian Y., Ballington J., Raja A., Brouwer C., Reid R., Burke M., Wang X., Rowland L.J., Bassil N., Brown A. (2014). Patterns of simple sequence repeats in cultivated blueberries (*Vaccinium* section *cyanococcus* spp.) and their use in revealing genetic diversity and population structure. Mol. Breed..

[10] Manzanero B.R., Kulkarni K.P., Vorsa N., Reddy U.K., Natarajan P., Elavarthi S., Iorizzo M., Melmaiee K. (2023). Genomic and evolutionary relationships among wild and cultivated blueberry species. BMC Plant Biol..

[11] Lyrene P., Vorsa N., Ballington J. (2003). Polyploidy and sexual polyploidization in the genus *Vaccinium*. Euphytica.

[12] Moore J.N., Eck P., Childers N.F. (1966). Breeding. Blueberry Culture.

[13] Galletta G.J., Moore J.N., Janick J. (1975). Blueberries and cranberries. Advances in Fruit Breeding.

[14] Rousi A. (1963). Hybridization between *Vaccinium uliginosum* and cultivated blueberry. Ann. Agric. Fenn..

[15] Rousi A. (1966). Cytological observations on some species and hybrids of *Vaccinium*. Der Zücht..

[16] Dweikat I., Lyrene P. (1989). Production and evaluation of a synthetic hexaploid in blueberry. Theor. Appl. Genet..

[17] Dweikat I.M., Lyrene P. (1991). Induced tetraploidy in a *Vaccinium elliottii* facilitates crossing with cultivated highbush blueberry. J. Am. Soc. Hort. Sci..

[18] Chavez D.J., Lyrene P. (2009). Interspecific crosses and backcrosses between diploid *Vaccinium darrowii* and tetraploid southern highbush blueberry. J. Am. Soc. Hort. Sci..

[19] Ehlenfeldt M.K. (2021). Production of dwarfs in rabbiteye blueberry (*V. virgatum* Aiton) crosses. J. Am. Pomol. Soc..

[20] Lyrene P., Sherman W. (1983). Mitotic instability and 2*n* gamete production in *Vaccinium corymbosum* × *V. elliottii* hybrids. J. Am. Soc. Hort. Sci..

[21] Megalos B.S., Ballington J.R. (1988). Unreduced pollen frequencies versus hybrid production in diploid-tetraploid *Vaccinium* crosses. Euphytica.

[22] Vorsa N., Ballington J.R. (1991). Fertility of triploid highbush blueberry. J. Am. Soc. Hort. Sci..

[23] Norden E.H., Lyrene P., Chaparro J.X. (2020). Ploidy, fertility, and phenotypes of F_1_ hybrids between tetraploid highbush blueberry cultivars and diploid *Vaccinium elliottii*. HortScience.

[24] Ballington J.R. (2001). Collection, utilization, and preservation of genetic resources in *Vaccinium*. HortScience.

[25] Ballington J.R., Rooks S.D., Cline W.O., Meyer J.R., Milholland R.D. (1996). The North Carolina State University Blueberry breeding program-toward *V. Covilleanum*?. VI Int. Sym. Vac. Cult..

[26] Draper A., Hancock J. (2003). Florida 4B: Native blueberry with exceptional breeding value. J. Am. Pom. Soc..

[27] Lyrene P. (1988). Fecundity of crosses between tetraploid and hexaploid *Vaccinium*. J. Am. Soc. Hort. Sci..

[28] Chu Y., Lyrene P. (2025). Artificial induction of polyploidy in blueberry breeding: A review. HortScience.

[29] Lyrene P.M. (2011). First report of *Vaccinium arboreum* hybrids with cultivated highbush blueberry. HortScience.

[30] Lyrene P.M. (2016). Florida native blueberries and their use in breeding. Acta Hortic..

[31] Lyrene P.M., Olmstead J.W. (2012). The use of inter-sectional hybrids in blueberry breeding. Int. J. Fruit Sci..

[32] Lyrene P.M. (2013). Fertility and other characteristics of F_1_ and backcross_1_ progeny from an intersectional blueberry cross [(highbush cultivar× *Vaccinium arboreum*) × highbush cultivar]. HortScience.

[33] Marangelli F., Pavese V., Vaia G., Lupo M., Bashir M.A., Cristofori V., Silvestri C. (2022). In vitro polyploid induction of highbush blueberry through de novo shoot organogenesis. Plants.

[34] Lei L., Liu G., Yan D., Zhang M., Cui Q., Zhao Q., Chu L., Wen L., Wang L., Du Q. (2023). Manipulation of ploidy for blueberry breeding: In vitro chromosome doubling of diploid *Vaccinium duclouxii* (Lévl.) Hand.-Mazz by trifluralin. Sci. Hort..

[35] Lineberger R.D. (1983). Origin, development, and propagation of chimaeras. Foliage Dig..

[36] Geier T. (2012). Chimeras: Properties and dissociation in vegetatively propagated plants. Plant Mutation Breeding and Biotechnology.

[37] Dermen H. (1947). Periclinal cytochimeras and histogenesis in cranberry. Am. J. Bot..

[38] Coyner M.A., Skirvin R.M., Norton M.A., Otterbacher A.G. (2005). Thornlessness in Blackberries. Sm. Fruits Rev..

[39] Miyashita C., Ishikawa S., Mii M. (2009). In vitro induction of the amphiploid in interspecific hybrid of blueberry (*Vaccinium corymbosum* × *Vaccinium* ashei) with colchicine treatment. Sci. Hort..

[40] Eng W.-H., Ho W.-S. (2019). Polyploidization using colchicine in horticultural plants: A review. Sci. Hort..

[41] Zhou H.-w., Zeng W.-D., Yan H.-b. (2017). In vitro induction of tetraploids in cassava variety ‘Xinxuan 048’using colchicine. Plant Cell Tissue Organ Cult. (PCTOC).

[42] Roy A.T., Leggett G., Koutoulis A. (2001). In vitro tetraploid induction and generation of tetraploids from mixoploids in hop (*Humulus lupulus* L.). Plant Cell Rep..

[43] Chen L.-L., Gao S.-L. (2007). In vitro tetraploid induction and generation of tetraploids from mixoploids in *Astragalus membranaceus*. Sci. Hortic..

[44] Roux N., Dolezel J., Swennen R., Zapata-Arias F.J. (2001). Effectiveness of three micropropagation techniques to dissociate cytochimeras in *Musa* spp.. Plant Cell Tiss. Organ Cult..

[45] Lyrene P. (1980). Micropropagation of rabbiteye blueberries. HortScience.

[46] Frett J.J., Smagula J.M. (1983). In vitro shoot production of lowbush blueberry. Can. J. Plant Sci..

[47] Wolfe D., Eck P., Chin C.-K. (1983). Evaluation of seven media for micropropagation of highbush blueberry. HortScience.

[48] Chandler C.K., Draper A.D. (1986). Effect of zeatin and 2iP on shoot proliferation of three highbush blueberry clones in vitro. HortScience.

[49] Grout J.M., Read P.E. (1986). Influence of stock plant propagation method on tissue culture and leaf-bud propagation of ‘Northblue’ blueberry. J. Am. Soc. HortSci..

[50] Brissette L., Tremblay L., Lord D. (1990). Micropropagation of lowbush blueberry from mature field-grown plants. HortScience.

[51] Debnath S.C. (2004). In vitro culture of lowbush blueberry (*Vaccinium angustifolium* Ait.). Sm. Fruits Rev..

[52] Ostrolucká M.G., Libiaková G., Ondrußková E., Gajdoßová A. (2004). In vitro propagation of In vitro *Vaccinium* species *Vaccinium*. Acta Univ. Latv..

[53] Gajdosova A., Ostrolucká M.G., Libiaková G., Ondrušková E., Šimala D. (2006). Microclonal propagation of *Vaccinium* sp. and Rubus sp. and detection of genetic variability in culture in vitro. J. Fruit Ornam. Plant Res..

[54] Jain S.M., Häggman H. (2007). Protocols for Micropropagation of Woody Trees and Fruits.

[55] Ružić D., Vujović T., Libiakova G., Cerović R., Gajdošova A. (2012). Micropropagation in vitro of highbush blueberry (*Vaccinium corymbosum* L.). J. Berry Res..

[56] Vescan L.A., Pamfil D.O., Clapa D., Fira A.L., Sisea C.R., Pop I.F., Petricele I.A., Ciuzan O., Opo R. (2012). Efficient micropropagation protocol for highbush blueberry (*Vaccinium corymbosum* L.) cv.‘Elliot’. Roman. Biotechnol. Lett..

[57] Fan S., Jian D., Wei X., Chen J., Beeson R.C., Zhou Z., Wang X. (2017). Micropropagation of blueberry ‘Bluejay’ and ‘Pink Lemonade’ through in vitro shoot culture. Sci. Hort..

[58] Schuchovski C.S., Biasi L.A. (2019). In vitro establishment of ‘Delite’ rabbiteye blueberry microshoots. Horticulturae.

[59] Cappai F., Garcia A., Cullen R., Davis M., Munoz P.R. (2020). Advancements in low-chill blueberry *Vaccinium corymbosum* L. tissue culture practices. Plants.

[60] Kharel P., Creech M.R., Nguyen C.D., Vendrame W.A., Munoz P.R., Huo H. (2022). Effect of explant type, culture medium, and BAP concentration on in vitro shoot development in highbush blueberry (*Vaccinium corymbosum* L.) cultivars. Vitro Cell. Dev. Biol. Plant.

[61] Schuchovski C., Biasi L.A. (2022). Micropropagation of *Vaccinium virgatum* ‘Delite’: A rabbiteye cultivar adapted to mild winters. Plant Biosyst..

[62] Billings S.G., Chin C.K., Jelenkovic G. (1988). Regeneration of blueberry plantlets from leaf segments. HortScience.

[63] Dweikat I., Lyrene P. (1988). Production and viability of unreduced gametes in triploid interspecific blueberry hybrids. TAG.

[64] Callow P., Haghighi K., Giroux M., Hancock J. (1989). In vitro shoot regeneration on leaf tissue from micropropagated highbush blueberry. HortScience.

[65] Rowland L.J., Ogden L.E. (1992). Use of a cytokinin conjugate for efficient shoot regeneration from leaf sections of highbush blueberry. HortScience.

[66] NeSmith D.S. (2008). ‘Rebel’ southern highbush blueberry. HortScience.

[67] Lyrene P.M. (2008). ‘Emerald’ southern highbush Blueberry. HortScience.

[68] Debnath S.C. (2007). Propagation of *Vaccinium* in vitro: A review. Int. J. Fruit Sci..

[69] Cao X., Hammerschlag F. (2000). Improved shoot organogenesis from leaf explants of highbush blueberry. HortScience.

[70] Meiners J., Schwab M., Szankowski I. (2007). Efficient in vitro regeneration systems for *Vaccinium* species. Plant Cell Tissue Organ Cult. (PCTOC).

[71] Chen H.-Y., Liu J., Pan C., Yu J.W., Wang Q.C. (2018). In vitro regeneration of adventitious buds from leaf explants and their subsequent cryopreservation in highbush blueberry. Plant Cell Tissue Organ Cult. (PCTOC).

[72] Chavez D.J., Lyrene P. (2009). Production and identification of colchicine-derived tetraploid *Vaccinium darrowii* and its use in breeding. J. Am. Soc. Hort. Sci..

[73] Qiu D., Wei X., Fan S., Jian D., Chen J. (2018). Regeneration of blueberry cultivars through indirect shoot organogenesis. HortScience.

[74] Sattler M.C., Carvalho C.R., Clarindo W.R. (2016). The polyploidy and its key role in plant breeding. Planta.

[75] Goldy R.G., Lyrene P. (1984). In vitro colchicine treatment of 4x blueberries, *Vaccinium* sp.. J. Am. Soc. Hort. Sci..

[76] Kazemian M., Ghasemi Omran V.O., Mohajel Kazemi E., Kolahi M. (2021). Regeneration of pinwheel phenotype and evaluation of anthocyanin in African violet (*Saintpaulia ionantha Wendl*.) periclinal chimera. J. Plant Mol. Breed..

[77] Marcotrigiano M. (1986). Origin of adventitious shoots regenerated from cultured tobacco leaf tissue. Am. J. Bot..

[78] Liu C., Callow P., Rowland L.J., Hancock J.F., Song G. (2010). Adventitious shoot regeneration from leaf explants of southern highbush blueberry cultivars. Plant Cell Tissue Organ Cult. (PCTOC).

